# Dietary L-tryptophan supplementation alleviates ammonia-induced stress in Nile tilapia fingerlings

**DOI:** 10.1038/s41598-025-29133-9

**Published:** 2025-12-03

**Authors:** Eman Y. Mohammady, Amina Y. Kamal, Mohamed A. Elashry, Abdelkarim I. M. El-Sayed, Mohamed S. Hassaan

**Affiliations:** 1 Aquaculture Division, National Institute of Oceanography and Fisheries, NIOF, Cairo, Egypt; 2 Department of Animal Production, Fish Research Laboratory, Faculty of Agriculture at Moshtohor, Benha University, Benha, 13736 Egypt

**Keywords:** L-tryptophan, NH_4_Cl, Oxidative stress, Nile tilapia, Physiology, Zoology

## Abstract

L-tryptophan dietary supplementation is a useful tactic to increase aquatic animals’ energy stores and antioxidant capability, thereby enhancing their resistance to environmental stressors like ammonia stress. Therefore, the present study aims to investigate the influence of tryptophan supplementation on the growth in Nile tilapia as well as the protective efficiency of tryptophan on blood indices, immune response, and antioxidant status against ammonium chloride (NH_4_Cl) stress. Fish were fed levels of tryptophan diets (0, 2 g, 4 g, and 8 g/kg diets) for 70 days after which fish were exposed to total ammonia (TA-N) (0 and 5 mg/L; 0.35 NH_3_–N) for 10 days. The study found that higher dietary L-tryptophan levels led to a linear increase in final body weight (*P* = 0.031), weight gain (*P = 0.045*), and specific growth rate (*P* = 0.032) in Nile tilapia fish. However, dietary supplementation with L-tryptophan at 4 and 8 g/kg significantly improved various physiological parameters in fish, irrespective of ammonia (NH_4_Cl) exposure. Notably, the 8 g/kg dosage yielded the most pronounced benefits (*P* < 0.05). L-tryptophan enhanced hematological health by elevating hemoglobin, hematocrit, red blood cells, white blood cells, and lymphocytes (*P* < 0.05). It also alleviated NH_3_–N stress by lowering liver enzymes (alanine aminotransferase, aspartate aminotransferase, alkaline phosphatase), cortisol, and glucose levels, while improving total protein, albumin, globulin, and nitric oxide concentrations (*P* < 0.05). Additionally, hepatic antioxidant status improved, as seen by increased superoxide dismutase and glutathione levels, with the highest levels recorded in fish fed on the 8 g L-tryptophan/kg diet and exposed to ammonia, and reduced malondialdehyde content (*P* < 0.05). Moreover, L-tryptophan supplementation enhanced immune responses in fish exposed to NH_3_-N, as indicated by increased levels of complement component (C3 and C4), and IgM (*P* < 0.05). These results suggested that L-tryptophan (8 g/kg diet) plays a protective and enhanced role in the growth, immunity, and antioxidant status of Nile tilapia under stress conditions such as ammonia exposure.

## Introduction

As the global population continues to grow, the demand for additional food sources is becoming increasingly urgent^1,2^. Aquaculture has emerged as a promising solution, providing a consistent supply of nutritious animal protein while offering substantial economic benefits^3^. However, the rising demand for aquatic animals and the constraints on resources such as water, land, and infrastructure have led to a shift toward more intensive aquaculture practices^4^. But the intensive systems effectively boost production, they often negatively impact the health and welfare of aquatic animals^5,6^. However, the aquaculture industry encounters numerous challenges associated with providing appropriate nutrition, managing environmental stress, and preventing disease. It is critical for the aquaculture industry to address current drawbacks and anticipate future challenges. Intensification of aquaculture systems may lead to increased fish sensitivity to environmental factors such heat and ammonia stress^7^.

The accumulation of waste, including uneaten feed and animal feces, in these systems contributes to increased ammonia levels in the water^8^. Ammonia, a byproduct of fish metabolism, exists in water as unionized ammonia (NH_3_-N) and ionized ammonium (NH_4_^+^-N), with their toxicity and concentrations influenced by temperature and pH^9^. In high-density aquaculture systems, NH_3_-N can quickly accumulate beyond safe levels, mainly due to overfeeding, poor water management, and insufficient filtration. This buildup can have serious harmful effects on osmoregulation, respiration, metabolism, bacterial infection, hematology, and the overall wellbeing of the farmed fish like common carp, Nile tilapia, and goldfish leading to neurotoxicity and oxidative stress^10–12^. Also, elevated ammonia levels weaken immunity, alter the expression of immune and inflammatory genes, increase mortality, and disrupt brain function of fish by interfering with amino acid neurotransmitter metabolism^13–15^.

Proposed strategies to mitigate ammonia toxicity include converting it into less harmful substances like urea and enhancing detoxification by minimizing proteolysis and amino acid breakdown^16^. Adding dietary supplements is an effective way to enhance the antioxidant capacity and energy reserves of aquatic animals, helping them cope better with environmental stress^17^. This strategy has gained significant attention in aquaculture for its practicality and benefits, attracting interest from both fish farmers and researchers. Among the various methods to boost production in intensive farming, functional feed additives stand out as a key approach^18^. Supplements like amino acids, vitamins, probiotics, medical plants seed extract, and rosmarinic acid have been widely studied for their role in improving fish performance, health, and immunity^15,19–21^. Amino acids are essential for metabolism and structure^22^. They are crucial for protein synthesis and serve as building blocks for enzymes, hormones, and antibodies, all of which are necessary to support physiological and immune functions^23–25^.

Tryptophan (Trp) is an essential amino acid necessary for protein synthesis and is the sole precursor of serotonin (5-HT), a neurotransmitter that also serves as a precursor to melatonin. Both serotonin and melatonin play key roles in stress reduction. As a readily available feed-grade product, Trp supplementation offers a practical and cost-effective method to improve both animal welfare and production efficiency^26^. Because of its role in serotonin and melatonin synthesis, Trp is regarded as a functional amino acid for stress management^27,28^. In teleost fish, Trp is found in lower amounts compared to other essential amino acids, which explains the relatively low dietary requirements observed in growth studies. For example, Nile tilapia requires 2.8 g of Trp per kg of feed^22^. Like other animals, fish require 10 essential amino acids in their diet^22^. Research has shown that Trp supplementation affects fish behavior by modulating serotonin signaling, leading to reduced aggression and alleviation of stress-induced anorexia in various teleost species^27,29,30^. As a critical component of serotonin synthesis, the availability of Trp determines its dose-dependent effects. A deficiency in Trp can lead to mood disorders, anxiety, aggression, stress, and abnormal eating behaviors^31–33^. Moreover, Trp supplementation has been shown to reduce plasma cortisol levels, as seen in rainbow trout (*Oncorhynchus mykiss*)^34^.

Tilapia farming has become increasingly popular worldwide due to its high market demand, economic importance, and the species’ impressive ability to withstand various environmental and biological stressors^35,36^. Given these advantages, optimizing nutritional strategies to enhance Nile tilapia’s resilience to stress is essential. However, limited research has been conducted on how ammonia buildup impacts the performance of Nile tilapia when fed diets enriched with tryptophan. This study addresses this gap by examining the effects of dietary tryptophan supplementation on growth, feed efficiency, and metabolic responses in Nile tilapia exposed to stress caused by ammonium chloride.

## Materials and methods

### Diets and design

In the current study isoprotein (346.50 g kg^− 1^) and isoenergetic (18.93 MJ kg^− 1^) basal diets were formulated (Table 1). L-tryptophan (from Sigma-Aldrich, Egypt) was added to the basal diet at concentrations of 0, 2, 4, and 8 g/kg diets for a period of 70 days, before ammonia exposure. After this feeding period, the fish were exposed to total ammonia (TA-N) at levels of 0 and 5 mg/L (equivalent to 0.35 mg/L NH_3_–N) for 10 days. The L-tryptophan levels were used according to^37^. The proximate chemical composition of the experimental diets is presented in Table 1 according to^38^. The diet ingredients were blended in a feed grinder until homogenous (Hobart Corporation, Troy, OH, USA) and thoroughly mixed with soybean oil. Distilled water was added to the premixed ingredients and homogenized until a dough like paste was formed. The dough was shaped into pellets using a manual noodle maker. The moist pellets were immediately dried at room temperature and after dried kept in cellophane bags and cooled at 4 °C until use.

**Table 1:** Formulation and proximate composition of the basal diets (g kg diet^− 1^, dry matter).

Ingredient	g kg diet^− 1^
Fish meal	90
Soybean meal 44%	430
Corn gluten meal 62%	120
Yellow corn 8.5%	230
Wheat bran 14%	60
Fish oil	50
Vitamins and minerals^1^	20
Proximate analysis	
Protein	346. 5
Lipid	61.75
Ash	45.32
Fiber	48.82
Nitrogen free extract (NFE)	533.21
Gross energy MJ kg^− 1^	18.93

^1^Vitamin and mineral mixture kg^− 1^ of a mixture contains 4800 I.U. Vit A, 2400 IU cholecalciferol (Vit. D), 40 g Vit E, 8 g Vit K, 4.0 g Vit B12, 4.0 g Vit B2, 6 g Vit B6, 4.0 g, Pantothenic acid, 8.0 g Nicotinic acid, 400 mg Folic acid, 20 mg Biotin, 200 gm Choline, 4 g Copper, 0.4 g Iodine, 12 g Iron, 22 g Manganese, 22 g Zinc, 0.04 g Selenium. Folic acid, 1.2 mg; niacin, 12 mg; d-calcium pantothenate, 26 mg; pyridoxine. HCl, 6 mg; riboflavin, 7.2 mg; thiamine. HCl, 1.2 mg; sodium chloride (NaCl, 39% Na, 61% Cl), 3077 mg; ferrous sulphate (FeSO_4_.7H_2_O, 20% Fe), 65 mg; manganese sulphate (MnSO_4_, 36% Mn), 89 mg; zinc sulphate (ZnSO_4_.7H_2_O, 40% Zn), 150 mg; copper sulphate (CuSO_4_.5H_2_O, 25% Cu), 28 mg; potassium iodide (KI, 24% K, 76% I). ^2^NFE (Nitrogen free extract) = 100−(crude protein + lipid + ash + fibre content). ^3^Gross energy was calculated using gross calorific values of 23.63, 39.52, and 17.2 kJ/g for protein, fat, and carbohydrate, respectively, according to^117^.

### Fish cultivation techniques

All fish techniques in the present investigation were carried out following the relevant guidelines and regulations of the National Institute of Oceanography and Fisheries (NIOF) (NIOF-AQ4-F-25-R-015). Uniform size of Mono-sex Nile tilapia fingerlings was obtained from El-Sahaba Hatchery in Egypt. After arriving at the fish laboratory of Agriculture Benha University, fish were acclimated for two weeks in a cement pond (4 × 2 × 1.25 m). During this period, commercial feed (30% crude protein and 18 MJ gross energy) was used to fed fish in the acclimation period at 3% of their body weight, split into three daily meals. After acclimation, the fish, averaging 1.5 ± 0.04 g, were randomly placed into 16 glass aquaria (210 L for each, filled 180 L de-chlorinated water), 20 fish in each aquarium to represent four treatments with four replicates for a 70-day trial. Fish were fed with 0 g (control), 2 g, 4 g, and 8 g L-tryptophan kg^− 1^ diets and unexposed to ammonia for 70-days. The water temperature was maintained between 26 and 28 °C with aeration, and 10% of the water was replaced daily with fresh water. Fish were weighed every 15 days to adjust feeding, and water quality was monitored daily. Following the feeding trial (70-days), two aquaria of each treatment’s fish were exposed to 5 mg/L of TA-N (0.35 mg/L NH_3_-N) for ten days, whereas the other two tanks’ fish were not. The ammonia level was used according to^39^.

Ammonium chloride (Sigma-Aldrich, Egypt) was added to a stock tank, and its concentration was adjusted based on water temperature and pH to maintain a total ammonia nitrogen (TA-N) level of 5 mg/L (0.35 mg/L NH_3_-N). The fish were subjected to ammonia stress for 10 days, with daily water changes and adjustments to ammonia levels to maintain stable conditions throughout the trial, following established protocols^40–42^.

To track the water quality, samples of each aquarium were taken on a regular basis. The dissolved oxygen (DO) and water temperature were measured using a portable oxygen meter (Jenway, London, UK). The pH values were measured using a Digital Mini-pH Meter (model 55, Fisher Scientific, Denver, CO, USA). Un-ionized NH_3_, nitrite, and nitrate were estimated according to^43^. During the experimental period, the photoperiod was measured as 12-hour day and 12-hour night. All the parameters’ measured values fell within the acceptable range for fish culture^43^, except the ammonia.

### Growth and feed efficiency

The number of fish in each tank was counted before and after the 70-day feeding trial. Growth performance and feed efficiency calculations are provided in the footnote of Table 2.

### Hemato-biochemical indices

After a 70-day feeding trial and 10-day ammonium chloride exposure, the fish were fasted for 24 h before blood sampling. Five fish per tank from each replicate were randomly selected and euthanized with clove oil (500 mg/L). Blood samples were drawn from the caudal vein, divided into two portions: one with 10% EDTA for hematological analysis including hematocrit (Htc), hemoglobin (Hb), red blood cells (RBCs) and white blood cells (WBCs). Htc was determined as described by^44^, Hb was measured using hemoglobin kits, which is a standardized procedure of the cyanomethemoglobin method, and the total count of WBCs was carried out by the indirect method^45^. The other portion of the blood sample was used without anticoagulant for serum extraction and biochemical analysis. The remaining part of each blood sample was allowed to clot overnight at 4 °C and then centrifuged for 10 min at 3000 rpm and the non-hemolyzed serum was collected and kept at −20 °C until needed. Serum enzymatic activities of alanine aminotransferase (ALT), alkaline phosphatase (ALP), and aspartate aminotransferase (AST) were measured^46^, glucose levels were assessed with standard kits, cortisol was measured by chemiluminescence, and nitric oxide (NO) levels were determined^47^. Total serum protein and albumin levels were measured^48,49^, with globulin calculated as the difference between total protein (TP) and albumin^50^.

### Immune responses

Serum immunoglobulin M (IgM) levels were measured using an ELISA kit from Cusabio (Wuhan, China). Complement components C3 and C4 were assessed using an immunoturbidimetric method from Zhejiang Yilikang Biotech Co. Lysozyme activity was determined using modified turbidimetric methods based on^51,52^.

### Assessments of liver and gills antioxidant activity

Liver and gill samples from five fish per replicate were collected and homogenized after anesthetizing the fish with 3-aminobenzoic acid ethyl ester (MS 222, 100 mg/L). The samples were then rinsed with ice-cold phosphate buffer (0.9 mL saline, pH 7.0 per 0.1 g tissue), and centrifuged at 3000 g for 10 min. Following established protocols^53^, the homogenates were centrifuged, and the supernatant was used to measure superoxide dismutase (SOD) activity. The procedure outlined in reference^54^ were modified for catalase (CAT) activity as follows: A mixture of 2.5 ml of phosphate buffer (pH 7.0), 2 ml of H_2_O_2_ solution and 0.5 ml of sample was added to each tube. The hydrogen peroxide (H_2_O_2_ 30 mM) was used as a substrate and the decrease in H_2_O_2_ concentration at 22 °C was measured spectrophotometrically at 240 nm for 1 min and expressed as specific activity (U/g protein). Malondialdehyde (MDA) concentration was assessed using the method from^55^, and glutathione (GSH) activity was evaluated based on^47^.

### Data analysis

Data were checked for normality and homogeneity before analysis, and the SAS ANOVA procedure^56^ was used to analyze the results. Polynomial contrasts were used to detect linear and quadratic effects of various dietary L-tryptophan levels on the growth performance and feed utilization. The level of significance adopted was 5%. Statistical package SAS^56^ was used for all statistical analysis response variables. A two-way (2 × 4) ANOVA was conducted to examine the effects of NH_3_-N and L-tryptophan levels, and their interaction, followed by post hoc Tukey test (*P* < 0.05)^57^to evaluate significant differences between treatment means.

## Results

### Growth indices

Table 2 presents the impact of different levels of dietary L-tryptophan on the growth and feed utilization of Nile tilapia before NH_3_-N exposure. As L-tryptophan levels in the diet increased, there was a steady improvement in final body weight (FBW), weight gain (WG), and specific growth rate (SGR), with significant linear increases (*P* = 0.031, *P* = 0.045, *P* = 0.032). No significant differences were observed in feed intake (FI) at varying L-tryptophan levels. However, higher dietary L-tryptophan improved feed conversion ratio (FCR) (*P* = 0.046) and protein efficiency ratio (PER) (*P* = 0.023). At the optimal desirability level of 0.95, the highest values for WG, SGR, PER, and FCR were 15.64, 3.43, 1.41, and 2.36, respectively, with the ideal L-tryptophan level estimated at 8 g/kg.

**Table 2:** Growth and feed utilization of nile tilapia, *Oreochromis niloticus* fed diets supplemented with different levels of L-tryptophan for 70 days.

	Experimental treatments				±SE	*P*-value
	Supplemented with different levels of L-tryptophan (g kg^− 1^ diet)					
	Control	2 g	4 g	8 g		Linear
Initial body weight (IBW; g fish^− 1^)	1.54	1.56	1.53	1.56	0.021	0.544
Final body weight (FBW; g fish^− 1^)	13.60^d^	15.90^c^	16.33^b^	17.20^a^	0.256	0.031
Weight gain (WG; g fish^− 1^)	12.06^d^	14.34^c^	14.80^b^	15.64^a^	0.341	0.045
Specific growth rate (SGR; % day^− 1^)	3.11^b^	3.32^ab^	3.38^a^	3.43^a^	0.031	0.032
Feed intake (FI; g fish^− 1^)	18.10^c^	21.37^b^	21.02^a^	22.05^a^	0.325	0.562
Feed conversion ratio (FCR)	1.50^a^	1.49^ab^	1.42^b^	1.41^b^	0.051	0.046
Protein efficiency ratio (PER)	2.14^bc^	2.16^b^	2.26^a^	2.28^a^	0.023	0.023

The values are presented as means of triplicate groups (*n* = 3).

Means followed by different letters in the same row are significantly different (*P <* 0.05).

Calculations were made as described by^118^: Weight gain (g)WG = final weight (g) – initial weight (g); Specific growth rate (SGR) = LnW2 – LnW1/t (days), Where, Ln = the natural log; W1 = initial fish weight, W2 = the final fish weight in grams and t = Period in days; FCR = Feed intake (g)/weight gain (g); Protein efficiency ratio (PER) = Weight gain (g)/protein ingested (g).

### Hemato- biochemical indices

The hematological parameters are presented in Table 3. Exposure of fish to NH_3_-N showed significantly lower levels of WBCs, Hb, RBCs, Hct, and lymphocytes compared to the unexposed groups, regardless of L-tryptophan supplementation. However, supplementation with L-tryptophan at 4 and 8 g/kg of diet significantly increased (*P* < 0.05) Hb, Hct, RBCs, and lymphocytes, regardless of the NH_3_-N exposure, with the highest levels observed in the 8 g/kg diet group. Additionally, tryptophan supplementation improved blood parameters in fish exposed to NH_3_-N, with the group receiving 8 g of L-tryptophan per kg of diet showing the highest levels of Hb, Hct, RBCs, WBCs, and lymphocytes compared to the un-supplemented and NH_3_-N -exposed group.

**Table 3:** Hematological parameters: hemoglobin (Hb %), hematocrit (Hct dL^− 1^), red blood cells count (RBCs), white blood cells count (WBCs), and lymphocytes of fish fed dietary supplemented with L-tryptophan and exposed to NH_3_-N for 10 days.

T	NH_3_-N (mgL^− 1^)	L-tryptophan (g kg^− 1^ diet)	Hematological parameters				
			Hb (%)	Hct (dL^− 1^)	RBCs (×10^− 6^ µl)	WBCs (× 10^3^ mm^− 3^)	lymphocytes %
**Individual treatments mean**							
***Without ammonia treatment***							
T1	0	0	7.88^d^	10.63^b^	2.13^d^	22.30^f^	74.73^e^
T2	0	2	8.18^c^	12.16^a^	2.66^c^	37.21^c^	93.20^c^
T3	0	4	9.00^b^	12.30^a^	3.00^b^	48.12^b^	94.73^b^
T4	0	8	9.40^a^	12.43^a^	3.43^a^	48.66^b^	95.93^a^
***After ammonia treatment***							
T5	0.35	0	6.50^f^	7.61^d^	1.80^e^	66.00^a^	93.60^bc^
T6	0.35	2	5.56^g^	9.04^c^	2.13^d^	30.20^e^	65.60^g^
T7	0.35	4	6.80^e^	9.43^c^	2.63^c^	32.63^d^	72.33^f^
T8	0.35	8	7.70^d^	10.43^b^	2.76^bc^	35.96^c^	81.23^d^
**Pooled SE**			0.08	0.19	0.08	0.67	0.37
	0		8.61^p^	11.88^p^	2.80^p^	39.07^q^	89.65^p^
	0.35		6.64^q^	9.13^q^	2.33^q^	41.20^p^	78.19^q^
		0	7.19^z^	9.12^z^	1.96^z^	44.15^r^	84.16^b^
		2	6.87^y^	10.60^x^	2.40^y^	33.70^z^	79.40^y^
		4	7.90^x^	10.86^x^	2.81^x^	40.37^y^	83.53^x^
		8	8.55^r^	11.43^r^	3.10^r^	42.31^x^	88.58^r^
**ANOVA (** ***P*** **-value)**							
NH_3_-N			< 0.0001	< 0.0001	< 0.0001	0.0019	< 0.0001
L-tryptophan			< 0.0001	< 0.0001	< 0.0001	< 0.0001	< 0.0001
NH_3_-N × L-tryptophan			0.0009	0.0724	0.3243	< 0.0001	< 0.0001

^**†**^Treatments means represent the average values of three aquaria per treatment. Post hoc Tukey test was conducted for individual means only if there was a significant interaction (ANOVA: *P* < 0.05). Means followed by the same letter are not significantly different.

^**‡**^Main effect means followed by the same letter are not significantly different at *P* < 0.05 by post hoc Tukey test; p and q for NH_3_-N and r, x, y and z for L-tryptophan levels. Hb: Hemoglobin; Htc: hematocrit; RBCs: red blood cell count; WBCs: white blood cells.

Table 4 shows that the levels of ALT, ALP, cortisol, and AST were significantly higher in fish exposed to NH_3_-N (*P* < 0.05), while TP, albumin, globulin, glucose, and nitric oxide levels were lower, regardless of the L-tryptophan effect. However, L-tryptophan supplementation (*P* < 0.05) reduced ALT, ALP, cortisol, and AST levels, and improved TP, albumin, globulin, and nitric oxide levels, regardless of NH_3_-N exposure. Fish exposed to NH_3_-N and fed a diet containing 8 g of L-tryptophan per kg showed higher levels of nitric oxide, albumin, total protein, and globulin as well as lower levels of AST, cortisol, and glucose, compared to the un-supplemented and NH_3_-N exposed group.

**Table 4:** Biochemical blood indices of fish fed dietary supplemented with L-tryptophan and exposed to NH_3_-N for 10 days.

T	NH_3_-N (mgL^− 1^)	L-tryptophan (g kg^− 1^ diet)	Biochemical blood indices								
			ALT (UL^− 1^)	AST(UL^− 1^)	ALP	TP (g L^− 1^)	Albumin (g L^− 1^)	Globulin (g L^− 1^)	Glucose (mg dl^− 1^)	Cortisol (ng mL^− 1^)	NO
**Individual treatments mean** ^**†**^											
***Without ammonia treatment***											
T1	0	0	23.00^b^	31.40^b^	78.56^a^	2.13^d^	1.10^cd^	1.03^bc^	515.66^b^	6.19^c^	9.17^e^
T2	0	2	19.10^d^	14.26^f^	69.60^b^	2.03^de^	1.32^b^	0.71^de^	381.33^e^	4.69^d^	15.06^c^
T3	0	4	17.70^e^	13.63^f^	55.76^d^	2.63^b^	1.95^a^	0.68^e^	231.50^g^	3.47^e^	24.16^b^
T4	0	8	16.23^f^	12.83^g^	53.86^e^	3.31^a^	1.95^a^	1.35^a^	113.63^h^	2.29^f^	28.30^a^
***After ammonia treatment***											
T5	0.35	0	39.13^a^	37.30^a^	52.00^f^	1.87^e^	0.95^f^	0.92^cd^	615.66^a^	10.86^a^	8.40^e^
T6	0.35	2	20.80^c^	25.06^c^	58.00^c^	1.99^de^	1.03^e^	0.96^c^	410.33^c^	7.45^b^	11.13^d^
T7	0.35	4	21.06^c^	22.86^d^	50.66^g^	2.01^de^	1.07^de^	0.94^c^	391.00^d^	4.60^d^	12.30^d^
T8	0.35	8	21.33^c^	18.50^e^	52.33^f^	2.34^c^	1.14^c^	1.19^ab^	362.33^f^	3.60^e^	14.86^c^
**Pooled SE**			0.23	0.22	0.40	0.06	0.01	0.07	2.08	0.11	0.45
**Means of the main effects** ^**‡**^											
	0		19.01^q^	18.03^q^	64.45^p^	2.52^p^	1.58^p^	0.94^p^	310.53^q^	4.16^q^	19.17^p^
	0.35		25.58^p^	25.93^p^	53.25^q^	2.05^q^	1.04^q^	1.01^p^	44.83^p^	6.63^p^	11.67^q^
		0	31.06^r^	34.35^r^	65.28^r^	2.01^y^	1.02^z^	0.98^x^	565.66^r^	8.52^r^	8.78^z^
		2	19.95^x^	19.66^x^	63.80^x^	2.01^y^	1.17^y^	0.83^x^	395.83^x^	6.07^x^	13.10^y^
		4	19.38c^x^	18.25^y^	53.21^y^	2.32^x^	1.51^x^	0.81^x^	311.25^y^	4.04^y^	18.23^x^
		8	18.78^y^	15.66^z^	53.10^y^	2.82^r^	1.55^r^	1.27^r^	237.98^z^	2.94^z^	21.58^r^
**ANOVA (** ***P*** **-value)**											
NH_3_-N			< 0.0001	< 0.0001	< 0.0001	< 0.0001	< 0.0001	0.3381	< 0.0001	< 0.0001	< 0.0001
L-tryptophan			< 0.0001	< 0.0001	< 0.0001	< 0.0001	< 0.0001	0.0001	< 0.0001	< 0.0001	< 0.0001
NH_3_-N × L-tryptophan			< 0.0001	< 0.0001	< 0.0001	< 0.0001	< 0.0001	0.0332	< 0.0001	< 0.0001	< 0.0001

^**†**^Treatments means represent the average values of three aquaria per treatment. Post hoc Tukey test was conducted for individual means only if there was a significant interaction (ANOVA: *P* < 0.05). Means followed by the same letter are not significantly different. ^**‡**^Main effect means followed by the same letter are not significantly different at *P* < 0.05 by post hoc Tukey test; p and q for NH_3_-N and r, x, y and z for L-tryptophan levels. ALT: alanine aminotransferase, AST: aspartate aminotransferase; ALP, Alkaline phosphatase; TP: total protein; NO: nitric oxide.

### Immune responses

C3, IgM, C4, and lysozyme levels were lower in fish exposed to NH_3_-N, regardless of the L-tryptophan supplementation (Table 5). However, when L-tryptophan was added to the diet, the levels of these components increased, with the highest levels observed in fish fed a diet containing 8 g of L-tryptophan per kg, regardless of NH_3_-N exposure. Additionally, L-tryptophan supplementation boosted the levels of C3, C4, and IgM in fish exposed to NH_3_-N, with the highest levels recorded in the group fed the 8 g L-tryptophan/kg diet.

**Table 5:** Serum immune responses: complement component (C3), complement component (C4), immune Globulin M (IgM), and lysozyme of fish dietary supplemented with L-tryptophan and exposed to NH_3_-N for 10 days.

T	NH_3_-N (mgL^− 1^)	L-tryptophan (g kg^− 1^ diet)	Serum immune responses			
			C3	C4	IgM	Lysozyme
**Individual treatments mean**						
***Without ammonia treatment***						
T1	0	0	70.63^f^	12.20^e^	44.06^d^	1.073^e^
T2	0	2	86.56^c^	17.96^c^	47.76^c^	1.84^c^
T3	0	4	92.43^b^	21.50^b^	56.43^a^	2.28^b^
T4	0	8	102.73^a^	27.43^a^	57.93^a^	2.73^a^
***After ammonia treatment***						
T5	0.35	0	60.10^g^	9.40^f^	31.20^f^	0.84^f^
T6	0.35	2	76.83^e^	11.16^e^	40.26^e^	1.17^e^
T7	0.35	4	78.26^e^	14.00^d^	47.53^c^	1.85^c^
T8	0.35	8	82.33^d^	19.03^c^	51.43^b^	1.53^d^
**Pooled SE**			0.84	0.43	0.67	0.044
	0		88.09^p^	19.77^p^	51.55^p^	1.98^p^
	0.35		74.38^q^	13.40^q^	42.60^q^	1.34^q^
		0	65.36^z^	10.80^z^	37.63^z^	0.95^y^
		2	81.70^y^	14.56^y^	44.01^y^	1.50^x^
		4	85.35^x^	17.75^x^	51.98^x^	2.06^r^
		8	92.53^r^	23.23^r^	54.68^r^	2.13^r^
**ANOVA (** ***P*** **-value)**						
NH_3_-N			< 0.0001	0.5236	< 0.0001	< 0.0001
L-tryptophan			< 0.0001	0.4755	< 0.0001	< 0.0001
NH_3_-N × L-tryptophan			0.0006	0.0002	0.0020	< 0.0001

^**†**^Treatments means represent the average values of three aquaria per treatment. Post hoc Tukey test was conducted for individual means only if there was a significant interaction (ANOVA: *P* < 0.05). Means followed by the same letter are not significantly different.

^**‡**^Main effect means followed by the same letter are not significantly different at *P* < 0.05 by post hoc Tukey test; p and q for NH_3_-N and r, x, y and z for L-tryptophan levels.

### Hepatic antioxidant activities

In fish exposed to NH_3_-N, hepatic activities of catalase (CAT), glutathione (GSH), and superoxide dismutase (SOD) were significantly reduced (*P* < 0.05), while malondialdehyde (MDA) levels were elevated, regardless of whether their diets were supplemented with L-tryptophan (Table 6). However, the addition of L-tryptophan improved the activity of these hepatic enzymes, with the highest levels seen in fish fed diet containing 8 g of L-tryptophan per kg, regardless of NH_3_-N exposure. Increasing tryptophan levels also enhanced SOD and GSH activity, with the highest levels recorded in fish fed on the 8 g L-tryptophan/kg diet and exposed to NH_3_-N, which also showed the lowest MDA levels.

**Table 6:** Hepatic oxidative response (U/g protein) of fish dietary supplemented with L-tryptophan and exposed to NH_3_-N for 10 days.

T	NH_3_-N (mgL^− 1^)	L-tryptophan (g kg^− 1^ diet)	Antioxidant enzymes			
			SOD	CAT	GSH	MDA
**Individual treatments mean**						
***Without ammonia treatment***						
T1	0	0	65.93^g^	648.30^a^	95.24^e^	232.867^d^
T2	0	2	101.66^f^	471.00^a^	101.10^d^	103.500^f^
T3	0	4	182.16^a^	902.70^a^	154.24^c^	94.100^g^
T4	0	8	152.33^b^	1003.00^a^	173.44^b^	85.633^h^
***After ammonia treatment***						
T5	0.35	0	52.43^h^	1244.70^a^	55.01^g^	337.743^a^
T6	0.35	2	112.10^e^	361.10^a^	73.08^f^	286.473^b^
T7	0.35	4	114.88^d^	391.30^a^	183.30^a^	256.603^c^
T8	0.35	8	120.96^c^	419.20^a^	184.66^a^	163.433^e^
**Pooled SE**			0.61	330.19	0.78	0.91
	0		125.52^p^	756.3^p^	131.01^p^	129.02^q^
	0.35		100.09^q^	604.1^p^	124.01^q^	261.06^p^
		0	59.18^z^	946.5^r^	75.12^z^	285.30^r^
		2	106.88^y^	416.1^r^	87.09^y^	194.98^x^
		4	148.52^r^	647.0^r^	168.77^x^	175.35^y^
		8	136.65^x^	711.1^r^	179.05^r^	124.53^z^
**ANOVA (** ***P*** **-value)**						
NH_3_-N			< 0.0001	0.5236	< 0.0001	< 0.0001
L-tryptophan			< 0.0001	0.4755	< 0.0001	< 0.0001
NH_3_-N × L-tryptophan			< 0.0001	0.2959	< 0.0001	< 0.0001

^**†**^Treatments means represent the average values of three aquaria per treatment. Post hoc Tukey test was conducted for individual means only if there was a significant interaction (ANOVA: *P* < 0.05). Means followed by the same letter are not significantly different.

^**‡**^Main effect means followed by the same letter are not significantly different at *P* < 0.05 by post hoc Tukey test; p and q for NH_3_-N and r, x, y and z for L-tryptophan levels. SOD: superoxide dismutase; CAT: catalase; GSH: glutathione; MDA: malondialdehyde.

### Gills antioxidant enzymes activities

**Fig. 1:**
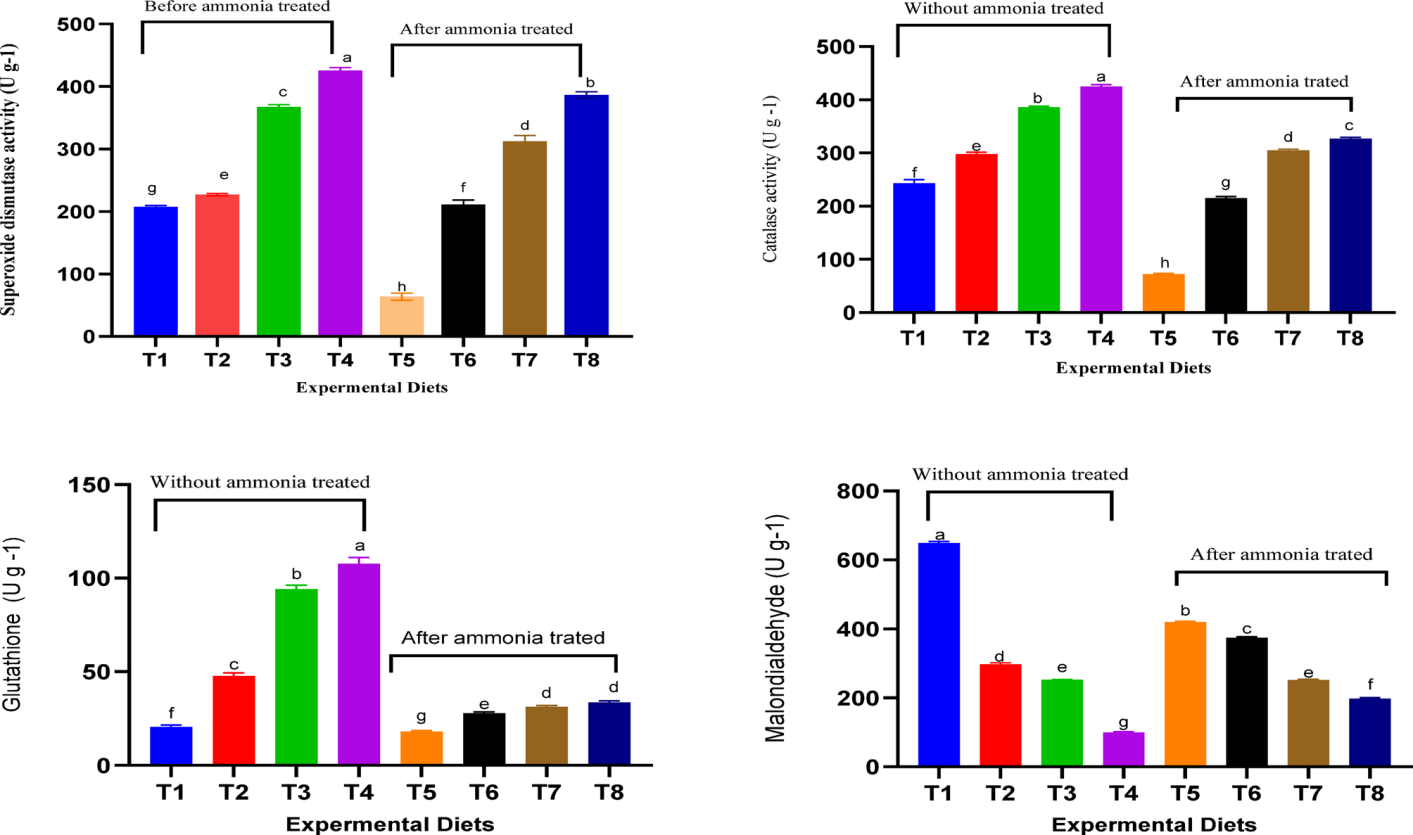
Gills oxidative response (U/g protein) of fish fed dietary supplemented with L-tryptophan and exposed to NH_3_-N for 10 days. T1 = (0 mgL^− 1^ NH_3_-N + 0 g L-tryptophan kg^− 1^ diet); T2 = (0 mgL^− 1^ NH_3_-N + 2 g L-tryptophan kg^− 1^ diet); T3 (0 mgL^− 1^ NH_3_-N + 4 g L-tryptophan kg^− 1^ diet); T4 = (0 mgL^− 1^ NH_3_-N + 8 g L-tryptophan kg^− 1^ diet); T5 (0.35 mgL^− 1^ NH_3_-N + 0 g L-tryptophan kg^− 1^ diet); T6 = (0.35 mgL^− 1^ NH_3_-N + 2 g L-tryptophan kg^− 1^ diet); T7 = (0.35 mgL^− 1^ NH_3_-N + 4 g L-tryptophan kg^− 1^ diet); T8 = (0.35 mgL^− 1^ NH_3_-N + 8 g L-tryptophan kg^− 1^ diet).

In fish exposed to NH_3_-N, the activities of catalase (CAT), glutathione (GSH), and superoxide dismutase (SOD), along with malondialdehyde (MDA) levels in the gills, showed a significant reduction (*P* < 0.05) irrespective of whether their diet was supplemented with L-tryptophan (Table 7; Fig. 1). Adding L-tryptophan to the diet increased the levels of CAT, GSH, and SOD, while reducing MDA levels, irrespective of NH_3_-N exposure. Fish fed a diet with 8 g of L-tryptophan per kg and not exposed to NH_3_-N exhibited the lowest MDA levels (*P* < 0.05) and the highest levels of CAT, GSH, and SOD. Similarly, fish that were exposed to NH_3_-N but fed the same L-tryptophan-enriched diet had lower MDA levels and higher CAT, GSH, and SOD activity compared to those not given the supplemented diet.

**Table 7:** ANOVA analysis of gills oxidative response (U/g protein) of fish fed dietary supplemented with L-tryptophan and exposed to NH_3_-N for 10 days.

T	NH_3_-N (mgL^− 1^)	L-tryptophan (g kg^− 1^ diet)	Antioxidant enzymes			
			SOD	CAT	GSH	MDA
**ANOVA (** ***P*** **-value)**						
NH_3_-N			< 0.0001	< 0.0001	< 0.0001	< 0.0001
L-tryptophan			< 0.0001	< 0.0001	< 0.0001	< 0.0001
NH_3_-N × L-tryptophan			< 0.0001	< 0.0001	< 0.0001	< 0.0001

SOD: superoxide dismutase; CAT: catalase; GSH: glutathione; MDA: malondialdehyde.

## Discussion

Studies have demonstrated that high inclusion levels of soybean meal (SBM), especially around 45%, can negatively impact growth performance and nutrient utilization in various fish species^6,58–60^. In this study, supplementing tilapia diets containing a high level of SBM (49%) with L-tryptophan enhanced feed and growth efficiency. This was evident from the improved weight gain rate (WGR), specific growth rate (SGR), and a lower feed conversion ratio (FCR). These benefits likely stem from L-tryptophan’s role in enhancing digestion, absorption, and protein synthesis^37^. Both^61,62^ reported that adding tryptophan to the diet enhanced growth and feed efficiency in juvenile GIFT tilapia, especially when fed plant-based diets. Similarly, studies on various fish species, including silver catfish, hybrid catfish, and rainbow trout, have shown that tryptophan supplementation can improve growth, digestion, and nutrient absorption. It also helps reduce feed conversion ratio and enhances protein efficiency, particularly in tilapia^63–65^. In meagre (*Argyrosomus regius*), mrigal fingerlings (*Cirrhinus mrigala*), and rohu, 0.25%, 1.36%, and 1.28% to 2.56% dietary tryptophan enhanced growth performance^66–68^. Silver barb also benefited from 0.4% tryptophan supplementation^69^. However, fish need a proper balance of all essential amino acids for healthy growth, and a deficiency in one like tryptophan can disrupt protein synthesis and lead to the breakdown of other amino acids into ammonia in liver^70^. As one of the most limiting amino acids after lysine and methionine, tryptophan is vital not only for protein formation but also to produce serotonin, which affects stress, behavior, appetite, and overall metabolism in fish^69,71^.

Ammonia buildup is a major issue in aquaculture, posing serious risks to the health and performance of aquatic animals^72^. This study is the first to examine how dietary L-tryptophan levels influence blood health, immunity, and antioxidant responses in Nile tilapia under ammonia stress, using blood and plasma markers as key indicators of overall fish health. The present results showed that hemoglobin (Hb), hematocrit (Htc), red blood cell count (RBCs), and lymphocyte levels significantly decreased in tilapia exposed to ammonia, regardless of L-tryptophan supplementation. These results are consistent with earlier studies conducted on different species, including tilapia^73^. Ammonia and other toxic substances are known to suppress erythropoiesis and hemoglobin synthesis, leading to reductions in RBCs, Hb, and Htc. Additionally, chemical exposure can cause blood extravasation, further reducing RBC levels^74^. Similar effects have been observed with pollutants like chromium, which can destroy mature RBCs and inhibit erythrocyte production in *Oreochromis* aureus^75^. L-tryptophan, an essential amino acid, has been shown to reduce stress in various farmed fish species by helping them cope with environmental and physiological challenges like temperature shifts, poor water quality, crowding, handling, ammonia, and low oxygen levels^7,28,76,77^. The current study showed that tilapia exposed to ammonia and treated with tryptophan improved blood health and recovery, with red blood cells, hematocrit, lymphocyte, and hemoglobin levels similar to those of the control group. Likewise, research by^78^ found that adding arginine to channel catfish diets positively affected their blood parameters, including hematocrit, hemoglobin, and red blood cells. Also^65^, highlighted that an increased red blood cell count is a sign of better fish health. In this study, L-tryptophan supplementation increased white blood cell (WBC) counts, countering the decrease caused by ammonia exposure. This suggests that L-tryptophan may boost fish health by enhancing the immune system’s resilience against stress and infections. L-tryptophan serves as a precursor for polyamines, which are vital for cell growth and differentiation.

Biochemical markers like albumin, globulin, total protein, and glucose were also examined to assess the impact of environmental stress on fish physiology. Total protein levels in fish serum are valuable indicators of liver function, blood osmolality, and kidney health, making them useful for detecting toxicity^79,80^. A significant decrease in total protein was observed in ammonia-stressed fish compared to the control group, likely due to increased protein breakdown triggered by corticosteroid hormones. These hormones facilitate protein breakdown and gluconeogenesis to meet the heightened energy demands during stress. This aligns with findings from studies on African catfish exposed to ochratoxin, *Cirrhinus cirrhosus* and *Gibelion catla* under ammonia stress, and Nile tilapia exposed to cadmium. However, fish in this study that were fed L-tryptophan enriched diets showed increased total protein levels and better tolerance to ammonia stress than those on un-supplemented diets.

Blood glucose plays a critical role in supplying energy to cells and tissues and serves as a reliable marker of environmental stress. When stress occurs, adrenaline is released, prompting the liver to break down glycogen stores, which raises blood glucose levels^81^. This makes blood glucose a key indicator for assessing stress and its physiological impacts^81–83^. In this study, tilapia exposed to ammonia showed a significant increase in serum glucose levels compared to the control group. This rise is associated with ammonia triggering the release of catecholamines, which in turn stimulates glycogen breakdown and gluconeogenesis, leading to higher glucose levels in the blood. These findings align with earlier research by^84^, who observed elevated glucose in stressed tilapia due to corticosteroid activity. Similarly^85,86^, reported increased plasma glucose in channel catfish and Nile tilapia exposed to high ammonia levels.

Other studies have found similar stress-induced glucose spikes in red drum^87^, seabream^88^, and Atlantic salmon^89^. Stress, whether from ammonia or other contaminants, can disrupt glucose metabolism, affect digestion, absorption, and utilization^73^. Interestingly, dietary L-tryptophan in this study reduced cortisol and glucose levels in tilapia, likely by inhibiting cortisol release. This suggests that L-tryptophan effectively mitigates ammonia stress in fish. Previous research also supports tryptophan’s stress-reducing effects in species like *C. mrigala* and *C. carpio*^23,90^. In *T. macdonaldi*, 1.55% dietary tryptophan prevented glucose increases during acute stress, while 0.99% and 1.55% reduced glucose levels under chronic stress^77,91^. Similarly, higher dietary tryptophan levels decreased glucose in *Labeo rohita* fingerlings exposed to temperature stress^76^.

Cortisol is widely recognized as a key indicator of stress in fish, with levels rising in response to stressors^23,90,92^. In this study, the highest serum cortisol levels were observed in groups exposed to NH_3_-N without dietary L-tryptophan, highlighting the stress induced by ammonia. Interestingly, as dietary L-tryptophan levels increased, serum cortisol levels in NH_3_-N exposed fish decreased, suggesting that L-tryptophan alleviates ammonia-related stress in tilapia. This aligns with earlier studies by^34^ on *Oncorhynchus mykiss*^23^, on *C. mrigala*, and^90^ on *C. carpio*, which reported that tryptophan-enriched diets reduced stress-induced cortisol elevation. These findings imply that elevated cortisol typically leads to immunosuppression in fish, while dietary L-tryptophan supplementation could support immune health under stress. However^93^, presented a contrasting perspective, showing that in unstressed fish, tryptophan-supplemented diets significantly reduced cortisol levels but did not prevent stress-induced cortisol increases.

AST, ALT, and ALP are important enzymes that help assess fish health. Elevated AST and ALT levels in the blood often indicate liver or kidney damage due to stress or pollution, while ALP reflects immune and cellular activity^94–96^. Monitoring these enzymes provides a reliable way to evaluate fish welfare^97^. In this study, fish exposed to NH_3_-N showed significantly higher levels of AST and ALT compared to the control group, suggesting that ammonia toxicity may cause pathological changes in fish organs^98^. However, when L-tryptophan was included in the diet either alone or combined with ammonia exposure, AST, ALT, and ALP activities significantly decreased (*P* < 0.05). This suggests that L-tryptophan may positively influence the health and nutritional status of fish, potentially by stimulating cytokines that protect liver cells. These findings highlight that L-tryptophan reduces the stress-induced biochemical parameters in fish exposed to NH_3_-N compared to fish treated with NH_3_-N alone. Interestingly, similar results have been reported for rohu (*Labeo rohita*) fed a diet containing 13.5 g/kg of threonine, which showed reduced ALT and AST activities under stocking density stress but increased ALP activity^99^. Comparable findings were also observed in jian carp^100^. Tryptophan has a role in increasing serotonin synthesis, which regulates the hypothalamic-pituitary-internal axis in fish^101^. This aligns with findings by^23^, where increased dietary tryptophan in *Cirrhinus mrigala* under crowding stress led to lower cortisol and ALT levels after an 8-week feeding period.

Immunoglobulins like IgM and complement proteins play vital roles in both the specific and nonspecific immune responses of fish^102,103^. The complement system, a key component of innate immunity, is essential for processes like inflammation, phagocytosis, microbial destruction, breakdown of immune complexes, and antibody production^104^. The main contributors to this process are hepatocytes, serum complements, and macrophages, which are produced by liver cells^105,106^. Stress responses, however, can suppress complement components like C3 and C4^107^. Lysozyme is a key enzyme in fish innate immunity that helps protect against bacterial infections by breaking down bacterial cell walls. It also supports immune defense by promoting phagocytosis and activating the complement system, making it an important marker of immune health^108^. In this study, exposure to NH_3_-N stress led to significant reductions (*P* < 0.05) in C3, IgM, lysozyme, and C4 levels, regardless of L-tryptophan supplementation. However, increasing dietary L-tryptophan in tilapia resulted in elevated levels of these immune components, with the highest value observed at 8 g/kg of L-tryptophan. This suggests that L-tryptophan enhances the nonspecific immune response in fish^109,110^, likely through its role in strengthening the complement system and maintaining homeostasis. Interestingly, a study on meagre (*Argyrosomus regius*) found no significant effect of dietary amino acid supplementation on lysozyme levels under stress from air exposure or crowding^111^. Further research is needed to fully understand how L-tryptophan influences fish serum IgM, C3, C4, and lysozyme levels under different stress conditions.

Under normal physiological conditions, aerobic organisms produce reactive oxygen species (ROS), which are balanced by the body’s ability to eliminate them^112^. When this balance is disrupted, oxidative stress occurs. In this study, oxidative stress enzymes such as catalase (CAT), superoxide dismutase (SOD), and glutathione (GSH) in the liver and gills were significantly elevated in response to NH_3_-N exposure. However, supplementation with L-tryptophan, with or without ammonium chloride exposure, effectively reduced the activity of these enzymes. This suggests that L-tryptophan plays a crucial role in the antioxidant defense system by scavenging free radicals, aligning with findings in juvenile grass carp and rohu (*Labeo rohita*) under threonine supplementation^99,100^. Additionally, L-tryptophan catabolism enhances immunity and exerts direct antioxidant and antimicrobial effects^22^. Cytokines released by immune cells can activate indoleamine 2,3-dioxygenase, an enzyme that breaks down L-tryptophan. This process reduces the availability of tryptophan for microbial biosynthesis. This enzyme, through the kynurenine pathway, also consumes superoxide anions, potentially reducing cellular antioxidant activity^22,37^. The reduced SOD and CAT activity observed in the high L-tryptophan group in this study may reflect this phenomenon. While limited data exists on the effects of dietary tryptophan on hepatic and gill antioxidant enzymes in tilapia under ammonia stress, studies on sea cucumbers (*Apostichopus japonicus*) show that dietary tryptophan increases SOD and CAT activity at low to medium stocking densities but decreases it at high densities. Malondialdehyde (MDA), a marker of oxidative damage from lipid peroxidation^113^, was also analyzed. In this study, MDA activity in the liver and gills decreased with L-tryptophan supplementation in both stressed and unstressed fish, suggesting a protective effect. MDA may act as a reservoir for damaged lipid molecules, like the function of threonine^114^. Similar findings have been reported in juvenile hybrid catfish^115^ and grass carp^116^, further supporting the role of L-tryptophan in mitigating oxidative damage in fish tissues.

## Conclusion

The current study demonstrates that dietary L-tryptophan is an effective feed supplement for Nile tilapia diets, particularly when added at 4 and 8 g/kg diet. The use of 4–8 g L-tryptophan/kg diets could also assist in reducing the negative effects of NH_3_-N toxicity, as evidenced by improved serum biochemical levels’ ability to combat NH_3_-N induced stress and oxidative/antioxidative biomarkers that suggested L-tryptophan’s tissue-protective properties. To demonstrate the protective effects of L-tryptophan treatment on fish against NH_3_-N toxicity in other fish, more thorough molecular studies are required, along with their modes of action and genomic paths.

## Data Availability

All data generated or analyzed during this study are included in this article. These data can also be made available from the corresponding author upon reasonable request.
